# The Role of miR-137 in Neurodegenerative Disorders

**DOI:** 10.3390/ijms25137229

**Published:** 2024-06-30

**Authors:** László Bodai, Roberta Borosta, Ágnes Ferencz, Mercédesz Kovács, Nóra Zsindely

**Affiliations:** 1 Department of Biochemistry and Molecular Biology, Faculty of Science and Informatics, University of Szeged, Közép fasor 52, H-6726 Szeged, Hungary; 2 Doctoral School of Biology, Faculty of Science and Informatics, University of Szeged, Közép fasor 52, H-6726 Szeged, Hungary; 3 Department of Genetics, Faculty of Science and Informatics, University of Szeged, Közép fasor 52, H-6726 Szeged, Hungary

**Keywords:** miRNA, microRNA, miR-137, neurodegeneration, Alzheimer’s disease, Parkinson’s disease, Huntington’s disease, amyotrophic lateral sclerosis, multiple sclerosis

## Abstract

Neurodegenerative diseases affect an increasing part of the population of modern societies, burdening healthcare systems and causing immense suffering at the personal level. The pathogenesis of several of these disorders involves dysregulation of gene expression, which depends on several molecular processes ranging from transcription to protein stability. microRNAs (miRNAs) are short non-coding RNA molecules that modulate gene expression by suppressing the translation of partially complementary mRNAs. miR-137 is a conserved, neuronally enriched miRNA that is implicated in neurodegeneration. Here, we review the current body of knowledge about the role that miR-137 plays in five prominent neurodegenerative disorders, including Alzheimer’s disease, Parkinson’s disease, Huntington’s disease, amyotrophic lateral sclerosis, and multiple sclerosis. The presented data indicate that, rather than having a general neuroprotective role, miR-137 modulates the pathology of distinct disorders differently.

## 1. Introduction

microRNAs (miRNAs) are a class of endogenous, short (~22 nucleotides) non-coding RNAs with a regulatory role [1]. miRNA encoding genes can be intergenic or they can be embedded in the introns or exons of protein-coding or non-coding genes [1,2]. Although the biogenesis of mature miRNAs from these genomic structures can follow different routes, the formation of most conserved miRNAs follows the canonic pathway, which involves the generation of a stem-loop structure containing primary transcript (pri-miRNA), followed by processing of this transcript by RNase III enzymes. During maturation, the sequences at the base of the stem-loop structure are released by the microprocessor complex in the nucleus, after which the resulting precursor miRNA (pre-miRNA) is exported to the cytosol, where another RNase III enzyme, Dicer, cleaves the loop structure generating an miRNA duplex [2]. One of the strands of this duplex, the guide strand, is then incorporated in the RISC ribonucleoprotein effector complex, which can regulate target genes by either translational repression or by cleavage of their mRNA products, depending on the degree of complementarity between the guide miRNA and the targeted mRNA [1,3]. miRNAs most commonly target the 3′-untranslated region (3′-UTR) of mRNAs, for translational repression, a seven-base pair complementarity between the miRNA 5′ seed region and the 3′-UTR of the targeted mRNA is sufficient [4,5]. Current annotations list 2654 mature miRNAs, each of which can target several mRNAs; therefore, it is not surprising that the number of human protein-coding genes regulated by miRNAs is predicted to be higher than 60%, meaning that miRNAs play a substantial role in the regulation of gene expression [6,7].

Neurodegenerative disorders are devastating diseases that substantially degrade the quality of life and often have fatal consequences. In some of the most common types of these disorders, such as Alzheimer’s disease (AD) and Parkinson’s disease (PD), age is a major risk factor; therefore, their prevalence and burden on society steadily increases as life expectancy increases globally [8,9,10]. Although the etiology of different neurodegenerative disorders varies, there are some similarities in their pathology, such as the accumulation of misfolded proteins or dysregulation of gene expression [11,12,13,14]. An increasing body of data indicates the role of miRNA-dependent post-transcriptional gene regulation in the pathogenesis of neurodegenerative diseases [15].

## 2. The Structure and Function of miR-137

**Figure 1:**
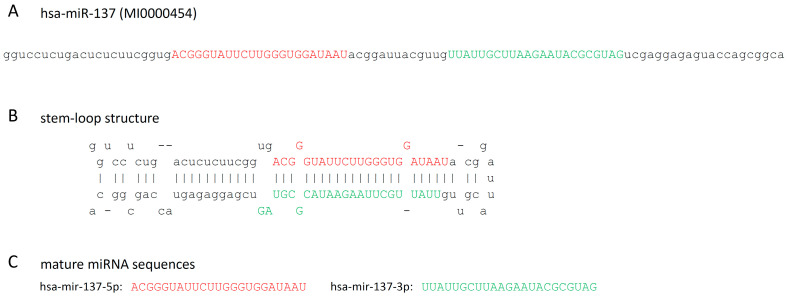
(**A**) The sequence of the hsa-miR-137 miRNA precursor, (**B**) its stem-loop structure, (**C**) and the sequence of the mature hsa-miR-137-5p and hsa-miRN-137-3p miRNAs [20]. Red and green colors mark the sequence of the -5p and -3p species, respectively.

miR-137 is a conserved microRNA, whose orthologs can be found in a variety of evolutionarily distant animals, including mouse (mmu-miR-137), *Drosophila* (dme-miR-137), and *C. elegans* (cel-miR-234) [16,17]. The human *MIR137* gene is located in the 1p21.3 (98046070-98046171 [-]) chromosomal position, embedded in the 61 kbp long non-coding RNA gene called the *MIR137 host gene* [6,18]. Its dominant mature miRNA product is the 23-nucleotide-long hsa-miR-137-3p (Figure 1), which gave 97% of the miR-137-specific sequence reads in the 71 sequencing experiments compiled by miRBase and has 1235 predicted gene targets in the miRDB database [19,20]. In contrast, the hsa-miR-137-5p species was represented by only 3% of miR-137-specific sequence reads and has only 21 predicted targets. These together suggest that miR-137-3p is the sole biologically relevant product of the *miR-137* gene. miR-137-3p was found to be highly expressed in neuroblastoma and adrenocarcinoma cells, and also in the adult brain, where the highest levels of expression were detected in the hippocampus and in cortical regions [6,20,21,22].

**Figure 2:**
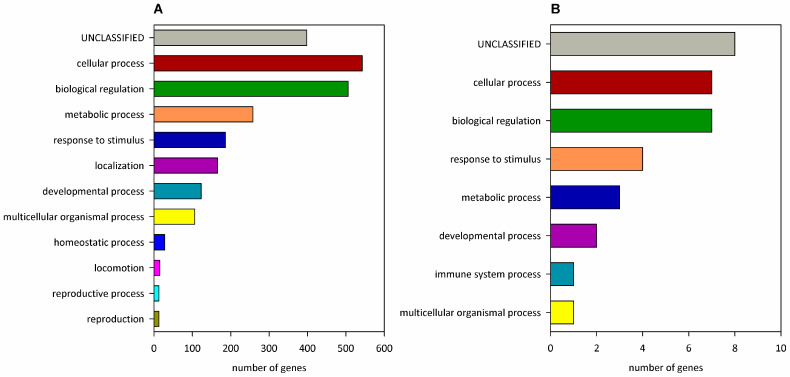
Classification of miR-137-regulated genes to biological processes. Predicted target genes of (**A**) hsa-miR-137-3p or (**B**) hsa-miR-137-5p [19] are classified into Panther v18.0 GO-Slim Biological Process categories. On panel A, GO categories with ≥10 genes are shown.

The targets of miR-137 are mostly involved in cellular, metabolic, and developmental processes, biological regulation, and response to stimulus (Figure 2). Some of the gene ontology biological process (GO BP) terms, in which miR-137-3p regulated genes are most over-represented (at FDR ≤ 0.001), are embryonic camera-type eye formation, regulation of activin receptor signaling pathway, ventricular cardiac muscle tissue morphogenesis, cardiac muscle cell differentiation, neuronal action potential, regulation of dendrite development, and protein dephosphorylation. miR-137-5p-regulated genes do not show significant enrichment in any GO BP category [23].

Several studies indicate that miR-137 plays a role in cell differentiation. In neuronal stem cells, the transcription of miR-137 is regulated by DNA methylation, the DNA methyl-CpG-binding protein MeCP2, and the transcription factor Sox2, which regulates stem cell renewal and neurogenesis [24]. During neuronal differentiation, miR-137 regulates the balance of neuronal stem cell proliferation and differentiation by downregulating epigenetic factors, such as the histone demethylase LSD1/KDM1A and the histone methyltransferase EZH2 and is also required for proper synaptogenesis and neuronal transmission [24,25,26]. Its effects are not limited to neurons, however, and it was also associated with the differentiation of embryonic stem cell-derived endothelial cells, differentiation of osteoblasts, and early erythroid commitment [27,28,29].

Due to its widespread regulatory roles, miR-137 was associated with several disorders, including various tumors, gestational diabetes, schizophrenia, and neurodegenerative disorders, the latter of which is in the focus of this review [30,31,32,33,34,35].

## 3. Alzheimer’s Disease

Alzheimer’s disease (AD) is the most prevalent form of dementia, whose hallmark features are amnestic impairment, hippocampal atrophy, and the presence of extracellular, amyloid beta-containing (Aβ) senile plaques and hyperphosphorylated tau protein-containing intracellular neurofibrillary tangles in the nervous system [36].

The expression level of miR-137 seems to show region-specific differences in the brains of AD patients. While ~five-fold downregulation was detected in the frontal cortex of sporadic AD patients [37], no change was reported from the analysis of post-mortem samples of the dorsolateral prefrontal cortex [38], the parietal lobe [39], the inferior frontal gyrus, the superior and middle temporal gyrus [40,41], the anterior temporal cortex, or the cerebellum [42]. Analysis of blood sera provided ambiguous results: some studies indicated that the level of miR-137 was downregulated in AD patients and in amnestic patients with mild cognitive impairment (MCI) [43,44], while other studies did not find a significant difference in the concentration of circulating miR-137 [45,46,47].

Dysregulation of miR-137 might influence AD pathology by altering the protein levels of its target genes, including CACNA1C (Calcium Voltage-Gated Channel Subunit Alpha1 C), PTN (Pleiotrophin), SPTLC1 (serine palmitoyltransferase long-chain base subunit 1), MAGL (monoacylglycerol lipase), USP30 (Ubiquitin-specific peptidase 30), and KREMEN1 (Kringle-Containing Transmembrane Protein 1) (Table 1), thereby modulating processes such as tau phosphorylation, apoptosis, mitochondrial, neuroinflammation, and the endocannabinoid system [37,44,48,49,50,51].

The dysregulation of miR-137 is influenced by altered expression of competing endogenous RNAs (ceRNAs) (Table 1). Two miR-137-targeting ceRNAs, small nucleolar RNA host gene 1 (SNHG1) and SNHG19 were identified in cell culture models of AD [48,55]. SNHG19 was upregulated in Aβ_25–35_-treated SH-SY5Y neuroblastoma cells, while its direct target, miR-137, was downregulated [55]. Aβ_25–35_ treatment also increased the expression of SNHG1 in SH-SY5Y and cultured human primary neuron cells, and its RNAi-mediated knock-down suppressed the negative effects of Aβ on both cell types [48]. Importantly, this coincided with the upregulation of miR-137-3p, which is one of its direct targets. The neuroprotective effects of SNHG1 knock-down on Aβ-induced phenomena were attenuated by miR-137 inhibitors or by overexpression of KREMEN1 (Kringle-Containing Transmembrane Protein 1), a direct target of miR-137-3p that functions as a Wnt antagonist, and was shown to contribute to synapse loss in a cortico-hippocampal transgenic murine cell culture model of AD [48,57]. These data indicate that SNHG1 upregulation contributes to Aβ pathology by weakening the miR-137-mediated suppression of KREMEN1.

Treatment of SH-SY5Y cells with Aβ_1–42_ peptide induces apoptosis, increases tau phosphorylation, and upregulates the protein level of the mitochondrial deubiquitinase USP30, a verified miR-137 target [44,51]. In this in vitro model, transfection of miR-137-5p mimics downregulated Aβ-induced apoptotic cell death and the level of tau phosphorylation [44,51]. The anti-apoptotic effects of miR-137-5p could be suppressed by overexpression of USP30, suggesting that miR-137-5p might influence Aβ pathology by downregulating USP30 [44]. This hypothesis was strengthened by observation in a chemically (D-galactose and AlCl_3_) induced murine model of AD, in which increased levels of Aβ_1-42_, USP30, and hyperphosphorylated tau proteins, atrophy of hippocampal and cortical neurons and spatial learning and memory deficits could be observed. Treatment with miR-137-5p agomir led to decreased Aβ_1-42_, USP30 and hyperphosphorylated tau levels, had a protective effect on hippocampal and cortical neurons, and ameliorated learning and memory problems. Overexpression of USP30 suppressed the positive effects of miR-137-5p on neuronal atrophy, learning, and memory, indicating that its effects are at least partially USP30-dependent [44].

High-fat diet is identified as a potential risk factor for AD [58,59], and human and animal studies indicate that miR-137 modulates high-fat diet-associated risk by directly regulating the expression of the SPTLC1 subunit of serine palmitoyltransferase, a key enzyme of sphingolipid biosynthesis [37]. The expression level of miR-137 was downregulated in primary rat astrocytes treated with palmitate and also in brain cortices and blood sera of wild-type mice fed a high-fat diet for 5 months [37,43]. In the cortex, this change coincided with increased expression of SPTLC1 and SPTLC2 proteins, but not their corresponding mRNAs, indicating a post-transcriptional regulatory effect [37]. In AD patients, analysis of cortical samples found elevated ceramide and sphingomyelin levels and increased amounts of SPTLC1 and SPTLC2 proteins, while miR-137 and miR-181c were downregulated [37]. Similarly, miR-137 was also downregulated in the cortices of transgenic TgCRND8 mice that express a double mutant form of APP 695 (carrying both the Swedish and the Indiana mutations, KM670/671NL1 and V717F, respectively) and were fed with high-fat chow for three months [60]. The regulatory relation between miR-137, SPTLC1, and Aβ was proven in primary astrocytes from TgCRND8 mice. In these cells, miR-137 and miR-181c overexpression led to reduced levels of endogenous Aβ and SPTLC1 protein, while co-transfection of SPTLC1 with miR-137/181c restored Aβ levels [37]. Thus, these data suggest that miR-137 protects against high-fat diet-related AD risk by antagonizing serine palmitoyltransferase expression that leads to the negative regulation of Aβ.

miR-137 also had a neuroprotective role in chemically induced rat AD models by modulating neuroinflammation and the endocannabinoid system [49,50]. miR-137 modulates neuroinflammation by antagonizing the expression of Pleiotrophin (PTN), a proinflammatory neurotrophic factor that is enriched in senile plaques in AD brains [50,61,62]. RNAi-mediated knock-down of PTN, a proven direct target of miR-137-3p, ameliorated increased neuronal apoptosis and damage of pyramidal cells in the hippocampus of rats treated with propofol, an intravenous anesthetic. Intriguingly, similar neuroprotective effects could be also achieved by miR-137 mimics in rat hippocampus and in human neuroblastoma cells where it coincided with normalization of the propofol-induced elevated levels of phosphorylated forms of PTN and its receptor, PTPRZ [50]. miR-137 also had a neuroprotective effect in a streptozotocin-induced rat AD model, which is characterized by memory impairment [49]. Overexpression of miR-137-3p or miR-let-7a in the hippocampal CA1 regions, in the central amygdala, or the medial prefrontal cortex of rats ameliorated streptozotocin-induced memory impairment while concurrently reducing the mRNA level of their direct target, the monoacylglycerol lipase-encoding *MAGL* [49]. Monoacylglycerol lipase is the main enzyme responsible for the hydrolysis of the endocannabinoid 2-arachidonylglycerol, whose expression was shown to be correlated with disease progression in the hippocampi of AD patients [63,64]. These results led to the suggestion that miR-137 can improve AD pathology by downregulating the degradation of endocannabinoids.

## 4. Parkinson’s Disease

Parkinson’s disease (PD) is the second most common neurodegenerative disorder after AD, which is characterized by loss of dopaminergic neurons in the substantia nigra, dysfunction of the basal ganglia, and intracellular aggregates of α-synuclein protein, called Lewy bodies. Although most cases are sporadic, more than 100 genetic loci were associated with susceptibility to PD [65]. 

The expression level of miR-137 was investigated both in neuronal tissues and blood plasma, but these studies provided ambiguous results. In the prefrontal cortex (Brodmann Area 9), miR-137 was downregulated in PD patients younger than 72.5 years, while there was no significant difference in older patients [66]. In contrast, the quantity of miR-137 was found to be unaltered in post-mortem substantia nigra samples of PD patients [67] and in the amygdala of advanced PD cases [68]. In the blood plasma of PD patients, the level of miR-137 was found to be elevated based on TaqMan low-density miRNA card measurement, but this result could not be verified on a larger cohort of 35 patients and 25 healthy controls by RT-qPCR [69]. A targeted RT-qPCR analysis of plasma levels of miR-137-3p in even larger cohorts of 60 sporadic patients and 60 controls found that mir-137-3p was upregulated in PD samples, but there was no association with accompanying depression and the severity of motor symptoms [70].

The level of miR-137-3p and the molecular processes it modulates can be affected by dysregulated ceRNAs, such as the imprinted long non-coding RNA (lncRNA) Opa-interacting protein 5 antisense RNA 1 (OIP5-AS1) that was downregulated in a 1-methyl-4-phenylpyridinium (MPP+)-induced SH-SY5Y cell model of PD [52] and is also implicated in a plethora of other disease conditions [71]. OIP5-AS1 binds miR-137-3p, and excess OIP5-AS1 depletes miR-137-3p in PD cells. In these cells, overexpression of OIP5-AS1 also results in increased viability and reduced expression of proinflammatory factors [52]. This positive effect, at least in part, can be attributed to the upregulation of the mitophagy receptor NIX, a direct target of miR-137-3p [52,72]. Upregulation of OIP5-AS1 was shown to deplete miR-137-3p in PD cells, which leads to the upregulation of NIX. Simultaneous addition of miR-137, however, negated this effect and led to NIX downregulation, resulting in increased ROS level and reduced mitochondrial membrane potential [52]. Therefore, the downregulation of OIP5-AS1 in PD cells might contribute to pathology by indirectly leading to NIX downregulation.

Results from chemically induced animal and cell culture models indicate that miR-137 has a negative impact on PD pathology by promoting oxidative stress and apoptosis. Oxidation resistance 1 (OXR1), a protein that provides protection against oxidative stress-induced DNA damage and neurodegeneration [73], is a direct target of miR-137-3p [53]. The level of OXR1 was found to be downregulated in a chemically induced (via 1-methyl-4-phenyl-1,2,3,6-tetrahydropyridine (MPTP) administration) mouse model of PD, and over-expressing it alleviated behavioral symptoms, neuronal apoptosis, and neuronal loss in the substantia nigra pars compacta of PD mice and reduced oxidative damage in an MPP+-induced primary neuronal cell culture PD model [53]. Treating PD neurons with PD mice serum-derived exosomes, which contain elevated levels of miR-137, decreased OXR1 levels, increased apoptosis, and oxidative damage, while simultaneous inhibition of miR-137 with an antagomir reversed these effects. Similarly, on the one hand, transfection of PD neurons with a miR-137 mimic promoted apoptosis, decreased the levels of OXR1 and Bcl-2 proteins, and increased the levels of 4-hydroxynonenal, cleaved-Caspase-3, and Bax; on the other hand, miR-137 inhibition had opposite effects [53]. These results argue that miR-137 has a negative effect on PD pathology by inhibiting OXR1 and thereby promoting oxidative stress and apoptosis. A supporting observation is that uric acid-primed mesenchymal stem cells (MSCs), in which miR-137-5p is downregulated, provide stronger neuroprotective effects (increased viability, reduced ROS generation, reduced levels of cleaved Caspase-3, cytochrome c, and Bax, and increased Bcl-2 levels) to MPP+-treated SH-SY5Y neuroblastoma cells in co-culture than control MSCs [74].

Investigations in *Drosophila* found that miR-137-3p was among the five miRNAs upregulated in a genetic disease model based on expression of α-synuclein A30P [75]. Notably, several direct targets of miR-137 that encode neurotransmitter receptors, such as the dopamine receptor encoding *D2R*, the metabotropic γ-aminobutyric acid receptor encoding *GABA-B-R3*, and the N-methyl-D-aspartate (NMDA) receptor encoding *Nmdar2,* were downregulated in PD flies, suggesting that miR-137 dysregulation might lead to faulty neurotransmission.

## 5. Huntington’s Disease

Huntington’s disease (HD) is a rare, inherited neurodegenerative disorder that most prominently causes the loss of striatal medium spiny neurons and leads to motor, cognitive, and psychiatric symptoms [76]. HD is caused by a dominant gene-of-function mutation in the *huntingtin* (*HTT*) gene that results in an aggregation-prone mutant huntingtin (Htt) protein with an abnormally long polyglutamine repeat [77]. As mutant Htt lies at the root of HD pathogenesis, factors that are able to modulate its production, stability, or aggregation might have a pivotal influence on pathology.

In HD, the most important role of miR-137 might be regulating the expression of *HTT* itself. *HTT* was found to be under the control of several miRNAs that regulate its translation, including miR-137-3p, mir-148a, and mir-214 [54]. miR-137-3p has an 8-mer binding site (a perfect seven-nucleotide match to positions 2–8 of the miRNA seed-sequence, followed by an adenine) at the 3′-UTR of huntingtin mRNA, which is highly conserved among vertebrates according to TargetScan predictions [54,78]. Experiments performed in HEK293T cells showed that overexpression of miR-137-3p led to decreased levels of endogenous huntingtin mRNA and protein [54]. Furthermore, miR-137-3p reduced the activity of a luciferase reporter gene construct with a single *HTT*-derived miR-137 binding site in HEK293T cells. These observations proved that miR-137-3p is indeed a negative regulator of *HTT* expression.

**Figure 3:**
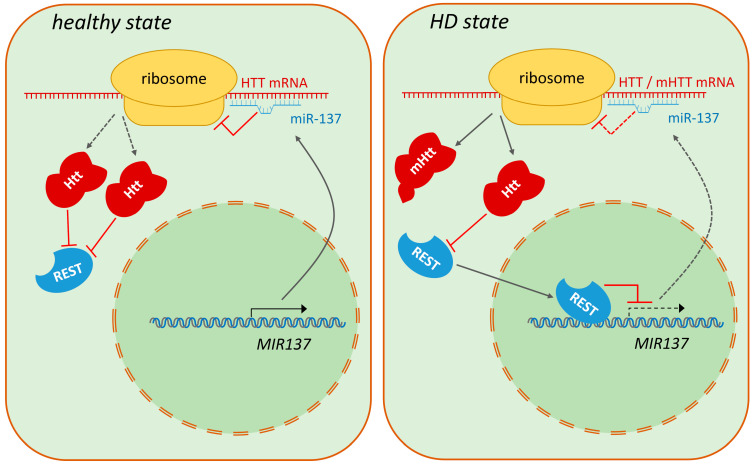
Model of the Htt—REST—miR-137 regulatory circuit in HD. In healthy individuals, wild-type Htt sequesters REST in the cytoplasm, thereby allowing higher levels of miR-137 expression, which leads to the suppression of Htt translation. In HD, mutant Htt loses its ability to inhibit the nuclear transfer of REST. REST represses the transcription of the *MIR137* gene, which results in lower miR-137 levels and consequent increase in the translation of the mutant Htt protein.

The level of miR-137 was analyzed in several brain regions in post-mortem patient samples. While significant changes in its abundance were not found in the prefrontal and frontal cortex [79,80], in dorsal caudal striatum samples of advanced (Vonsattel grade 4 [81]) HD patients miR-137 was among the 62 downregulated miRNAs [80]. This study also found that transcriptional targets of the REST (RE1-Silencing Transcription Factor) transcriptional repressor were enriched among the miRNAs downregulated in HD. REST is a known contributor to HD pathology that accumulates in the nucleus and represses its target genes in the disease state [82,83]. *MIR137* itself is a REST target [84], implying that its downregulation in the HD striatum, along with the downregulation of several other REST target miRNAs, might be the consequence of incorrect regulation of REST activity [80]. The connection between REST and miR-137 in HD pathology is further supported by experiments performed in the *Hdh^109/109^* mouse knock-in striatal cell culture model, which demonstrated that *miR-137* is a relevant direct transcriptional target of REST in HD. In *Hdh^109/109^* cells, miR-137 was shown to be significantly downregulated, which coincided with increased REST occupancy of the putative REST binding site (RE1) in the transcription regulatory region of *miR-137*. shRNA-mediated knock-down of REST in *Hdh^109/109^* cells resulted in upregulation of miR-137 to levels comparable to those measured in healthy control *Hdh^7/7^* cells [85]. The data presented above describe a regulatory circuit that involves Htt, REST, and mir-137 (Figure 3). In this, the presence of mutant Htt leads to increased nuclear translocation of REST and increased repression of REST target genes, including *miR-137*. Reduced miR-137-3p levels then result in increased translation of mutant Htt due to weaker negative control that can further aggravate pathology.

miR-137-3p was also significantly downregulated in a *Drosophila* model of HD, while its predicted targets were enriched among upregulated mRNAs, suggesting a causal link between the altered level of miR-137-3p and transcriptional dysregulation in the disease model. The putative contribution of miR-137 downregulation to HD pathology was supported by the observation that overexpression of miR-137 ameliorated several symptoms of HD flies including reduced lifespan and impaired motor activity [86]. Altered regulation of miR-137 was not detected in every animal model, however. A small-scale study analyzing changes in the miRNA transcriptome of several rodent models via miRNA microarray measurements did not find a significant change in miR-137 levels in striatal samples of a 3-nitropropionic acid-induced rat HD model, R6/2 mice, and YAC128 mice [87]. 

## 6. Amyotrophic Lateral Sclerosis

Amyotrophic lateral sclerosis (ALS) is a lethal neurodegenerative disorder that affects both the upper and lower motor neurons in the central nervous system. Around 20 genes have been associated with ALS; these are involved in molecular processes such as RNA metabolism, protein quality control, and axonal transport [88].

miR-137 was found to be dysregulated in a murine ALS model, but this finding was not corroborated in human subjects. In a genetic murine model of ALS, which is based on the low-level expression of a glycine-93 to alanine mutant form of human superoxide dismutase (G93A-SOD1), miR-137 was found to be upregulated in the spinal cord, and in the case of miR-137-3p, this change became more pronounced after longer disease progression [89]. In 95-day-old mice, corresponding to an earlier stage of the disease, both mmu-miR-137-5p and mmu-miR-137-3p were upregulated more than two-fold. In 108- and 122-day-old mice, corresponding to later disease stages, mmu-miR-137-3p was upregulated more than 13-fold and 8-fold, respectively, while there was no difference in the abundance of mmu-miR-137-5p based on microarray analysis [89].

Despite the results from the murine model, miR-137 was not among the dysregulated miRNAs identified in post-mortem spinal cord samples of sporadic ALS patients [90]. Similarly, miR-137 was not among the top 20 upregulated or downregulated miRNAs in post-mortem samples derived from the motor cortex of ALS patients [91].

miR-137 was also not among the dysregulated miRNAs in leukocytes isolated from the blood of Chinese [92] or Italian [93] sporadic ALS patients based on microarray analysis. It was also not identified among the differentially expressed miRNAs in peripheral blood [94,95] or serum, or plasma samples of sporadic ALS patients [96,97,98,99], in circulating small extracellular vesicles of ALS patients [100,101], and in skeletal muscle biopsies of ALS patients [102]. Microarray analysis did not find a significant change in miR-137 levels in blood serum samples of Caucasian patients having the familial form of the disease (having mutations in the *SOD1*, *FUS*, or *C9orf72* genes) either [103].

## 7. Multiple Sclerosis

Multiple sclerosis (MS) is an autoimmune disease, whose risk is influenced by both genetic and environmental factors. The pathological hallmark of MS is perivenular inflammatory lesions that lead to oligodendrocyte damage and demyelination and, finally, axonal damage [104]. miR-137 was not found among the miRNAs dysregulated in the white matter in MS [105,106]. However, bioinformatic analysis of data integrated from research articles, miRNA profiling datasets, and *in silico* predictions identified miR-137 as one of the members of an MS-specific miRNA regulatory network and found it to be significantly upregulated ~four-fold in blood sera of MS patients in validation experiments analyzing samples of 33 MS patients and 30 controls by RT-PCR [107]. Additionally, bioinformatic analysis identified two putative miR-137 target genes, *NDUFV3* (*NADH/ubiquinone oxidoreductase subunit V3*) and *C3orf38* (*chromosome 3 open reading frame 38*), that are deregulated in MS. Controversially, reversed changes were observed in another study analyzing sera samples of 108 MS patients and 104 controls [108]. In the MS cohorts, miR-137 was significantly downregulated ~25-fold, while the level of growth arrest-specific transcript (GAS5), a ceRNA targeting miR-137-3p, was upregulated two-fold, and MS risk was associated with specific variants of miR-137 and GAS5 [108]. Upregulation of GAS5 was shown to have a negative effect on neuronal survival in another study analyzing ischemic brain injury by repressing miR-137-3p, which consequently leads to the over-activation of the Notch1 signaling pathway [56]. 

## 8. Development of miR-137 Biosensors

Several research groups reported alteration in the levels of circulating miR-137 in neurodegenerative disorders, including AD, PD, and MS [43,44,69,70,107,108] (Table 2). Although these reports are often contradicted by other studies, the potential applicability of miR-137 as a biomarker in these disorders urged several research groups to develop sensitive methods for its detection. These nanobiosensors rely either on hybridization chain reaction (HCR) or on electrochemical detection and do not need RNA extraction and amplification.

The nanobiosensor developed by Delkhahi et al. combines HCR amplification with colorimetric detection of gold nanoparticles (AuNPs) into a sensitive (with detection limits of 0.25 nM and 10 nM in buffer and in serum, respectively) and selective enzyme-free method. In this, the presence of miR-137 leads to dsDNA formation that changes the aggregation property and spectrum of AuNPs [109]. Later, an even more sensitive enzyme- and label-free HCR-based nanobiosensor was developed for miR-137 detection with a sensitivity of 0.05 nM in buffer and 2 nM in serum. This nanobiosensor detects miR-137 via fluorescence measurement after a cascade of hybridization events between miR-137 and oligonucleotide probes that eliminates the quenching of SYBR Green I fluorescence by graphene oxide nanoparticles [110].

Electrochemical detection requires more specialized instrumentation but is even more sensitive. In these sensors, miR-137-specific antisense oligonucleotide capture probes were attached to gold nanowires [111], gold nanostar particles [112], or gold nanourchins [113] on the surface of the sensing electrode, and the change in the electrochemical properties after miR-137 hybridization was determined via voltammetry. These nanobiosensors are highly selective and can detect miR-137 in the fM concentration range, the most sensitive characterized by detection limits of 1.7 fM and 20 fM in buffer and human serum, respectively, and a linear quantitation range of 5 fM–750 fM [111].

## 9. Conclusions

The body of knowledge accumulated so far suggests that miR-137 is dysregulated in several neurodegenerative disorders and might be involved in their pathogenesis (Table 3). Its effects are not alike in different disorders, suggesting that it might rather play disease-specific than general neuroprotective roles. In ALS and MS, the role of miR-137 is not well supported or controversial. However, in AD, PD, and HD, modulating miR-137 levels can alter pathology. In AD, it has a positive effect by modulating Aβ levels, tau phosphorylation, mitochondrial function, neuroinflammation, the endocannabinoid system, and the effects of a high-fat diet. In contrast, miR-137 seems to have a negative effect on PD pathology by affecting mitochondria, oxidative stress, and apoptosis. Its most direct effect might be on HD pathology as it directly targets the HTT mRNA. These studies raise the possibility of the application of miR-137 agomirs and antagomirs as therapeutical agents and miR-137 biosensors for diagnostic purposes. Further research is necessary due to the low number of confirmatory studies and often small sample sizes.

## Figures and Tables

**Table 1:** Direct interacting partners of miR-137 identified in neurodegenerative disorders.

Abbreviation	Name	Biological Role ^1^	Interaction Effect	Interaction Implicated in ^2^	Ref.
CACNA1C	Calcium Voltage-Gated Channel Subunit Alpha1 C	subunit of a voltage-dependent L-type Ca^2+^ channel	downregulated by miR-137-3p	AD	[51]
MAGL	Monoacylglycerol lipase	hydrolyzes monoacylglycerides, including the brain endocannabinoid, 2-arachidonoylglycerol	downregulated by rno-miR-137	AD	[49]
PTN	Pleiotrophin	a neurotrophic factor involved in neural development and inflammation	downregulated by miR-137-3p	AD	[50]
SPTLC1	Serine palmitoyltransferase long-chain base subunit 1	subunit of serine palmitoyltransferase, a rate-limiting enzyme in de novo ceramide synthesis	downregulated by miR-137	AD	[37]
USP30	Ubiquitin-specific peptidase 30	mitochondrial deubiquitinase enzyme	downregulated by miR-137	AD	[44] ^3^
KREMEN1	Kringle-Containing Transmembrane Protein 1	Wnt antagonist, prevents glycogen synthase kinase-3 beta sequestration	downregulated by miR-137-3p	AD	[48]
NIX/BNIP3L	NIP3-Like Protein X/ Bcl-2/adenovirus E1B 19-kDa-interacting protein 3-like	mitophagy receptor	downregulated by miR-137-3p	PD	[52]
OXR1	Oxidation resistance 1	positively affects oxidative stress resistance, has protective effects against ROS	downregulated by miR-137-3p	PD	[53]
HTT	Huntingtin	affects vesicular trafficking, transcription, and apoptosis, mutated in HD	downregulated by miR-137-3p	HD	[54]
SNHG1	Small nucleolar RNA host gene 1	ceRNA	depletes miR-137-3p	AD	[48]
SNHG19	Small nucleolar RNA host gene 1	ceRNA	depletes miR-137-3p	AD	[55]
OIP5-AS1	Opa-interacting protein 5 antisense RNA 1	ceRNA	depletes miR-137-3p	PD	[52]
GAS5	Growth arrest-specific 5	ceRNA	depletes miR-137-3p	MS	[56]

^1^ ceRNA: Competing endogenous RNA. ^2^ AD: Alzheimer’s disease, PD: Parkinson’s disease, HD: Huntington’s disease, and MS: multiple sclerosis. ^3^ The referenced paper reports the interaction of the 3′-UTR of USP-30 and miR-137-5p, but the presented sequence corresponds to miR-137-3p.

**Table 2:** Expression of miR-137 in tissues of neurodegenerative disease patients.

Disease	Tissue	Expression Change ^1^
Alzheimer’s disease	frontal cortex [37]	downregulated
	dorsolateral prefrontal cortex [38]	NC
	parietal lobe [39]	NC
	inferior frontal gyrus [41]	NC
	superior temporal gyrus [40,41]	NC
	middle temporal gyrus [40]	NC
	anterior temporal cortex [42]	NC
	cerebellum [42]	NC
	whole blood [47]	NC
	plasma [46]	NC
	serum [43,44]	downregulated
	serum [45]	NC
Parkinson’s disease	prefrontal cortex [66]	downregulated (<72.5 years), NC (>72.5 years)
	substantia nigra [67]	NC
	amygdala [68]	NC
	plasma [69]	upregulated/NC
	plasma [70]	upregulated
Huntington’s disease	prefrontal cortex [79]	NC
	frontal cortex [80]	NC
	dorsal caudal striatum [80]	downregulated
Amyotrophic lateral sclerosis	spinal cord [90]	NC
	motor cortex [91]	NC
	skeletal muscle [102]	NC
	leukocytes [92,93]	NC
	whole blood [94,95]	NC
	plasma [98,101]	NC
	serum [96,97,99,100,103]	NC
Multiple sclerosis	white matter [105,106]	NC
	serum [107]	upregulated
	serum [108]	downregulated

^1^ NC: no change compared to healthy control.

**Table 3:** Pathomechanisms affected by miR-137 in neurodegenerative disorders ^1^.

Disease ^2^	Model	Pathomechanism Modifying Effects	Related Factors ^3^	References
AD	Aβ_25–35_-treated SH-SY5Y and HPN cells	miR-137-3p represses KREMEN1 and has a positive effect on pathology by contributing to increased cell viability, reduced apoptosis, and increased mitochondrial membrane potential.	SNHG1, SNHG19, KREMEN1	[48,55]
	Aβ_1–42_-treated SH-SY5Y cells and chemically induced AD mice	miR-137-5p reduces Aβ deposition, tau phosphorylation, and apoptosis by downregulating USP30.	USP30	[44,51]
	APP/PS1 double-transgenic AD mice	Downregulation of miR-137 coincides with upregulation of its target, CACNA1C.	CACNA1C	[51]
	AD patient cortex, TgCRND8 mice	miR-137 protects against high-fat diet-related AD risk by suppressing serine palmitoyltransferase expression that leads to lowered Aβ levels.	SPTLC1	[37,60]
	propofol-treated rats and SK-N-SH cells	miR-137-3p reduces neuronal apoptosis and restores cell proliferation by suppressing the proinflammatory neurotrophic factor PTN.	PTN	[50]
	streptozotocin-treated rats	miR-137-3p ameliorates memory impairment by downregulating MAGL (monoacylglycerol lipase), an endocannabinoid-degrading enzyme.	MAGL	[49]
PD	MPP+-induced SH-SY5Y cell model	Suppression of miR-137-3p by OIP5-AS1 leads to upregulation of the mitophagy receptor NIX, which contributes to reduced ROS levels and normalization of the mitochondrial membrane potential.	OIP5-AS1, NIX	[52]
	MPP+-induced rat primary neuronal cell	miR-137-3p decreases OXR1 levels and increases oxidative damage and apoptosis.	OXR1	[53]
	α-synuclein A30P-expressing *Drosophila*	Several neurotransmitter receptor targets of upregulated miR-137-3p are downregulated.	D2R, GABA-B-R3, Nmdar2	[75]
HD	HEK293T cells	miR-137-3p negatively regulates HTT translation.	HTT	[54]
	murine Hdh^109/109^ striatal cells	miR-137 is involved in REST-dependent transcriptional dysregulation.	REST	[85]

^1^ The table contains pathomechanisms that were experimentally explored/validated in the specific disorders. ^2^ AD: Alzheimer’s disease, PD: Parkinson’s disease, and HD: Huntington’s disease. ^3^ ceRNA names are underlined.

## Data Availability

Data supporting the content of this review can be found in the referenced research articles.
